# Digitization of Handwritten Chess Scoresheets with a BiLSTM Network

**DOI:** 10.3390/jimaging8020031

**Published:** 2022-01-30

**Authors:** Nishatul Majid, Owen Eicher

**Affiliations:** 1 Department of Physics and Engineering, Fort Lewis College, 1000 Rim Dr, Durango, CO 81301, USA; 2 Department of Computer Science, The Colorado School of Mines, 1500 Illinois St., Golden, CO 80401, USA; oeicher@mines.edu

**Keywords:** chess scoresheet recognition, offline handwriting recognition, convolutional bilstm network, latin handwriting recognition, handwritten chess dataset, chess moves digitization

## Abstract

During an Over-the-Board (OTB) chess event, all players are required to record their moves strictly by hand, and later the event organizers are required to digitize these sheets for official records. This is a very time-consuming process, and in this paper we present an alternate workflow of digitizing scoresheets using a BiLSTM network. Starting with a pretrained network for standard Latin handwriting recognition, we imposed chess-specific restrictions and trained with our Handwritten Chess Scoresheet (HCS) dataset. We developed two post-processing strategies utilizing the facts that we have two copies of each scoresheet (both players are required to write the entire game), and we can easily check if a move is valid. The autonomous post-processing requires no human interaction and achieves a Move Recognition Accuracy (MRA) around 95%. The semi-autonomous approach, which requires requesting user input on unsettling cases, increases the MRA to around 99% while interrupting only on 4% moves. This is a major extension of the very first handwritten chess move recognition work reported by us in September 2021, and we believe this has the potential to revolutionize the scoresheet digitization process for the thousands of chess events that happen every day.

## 1. Introduction

Chess is the one of the most popular board games in the world. Based on the surveys of the Agon Ltd. (2012), approximately 605 million adults play chess regularly [1], and this number has been exponentially rising since then. Although there are different variants of chess, most of the time it is played between two players. The first player plays with white pieces and the second player with black pieces. In a game, both players make their moves with their pieces in alternate turns until the game ends. During an Over-the-Board (OTB) chess event, both of these players for every match are generally required to record their own and their opponent’s moves on a chess move recording sheet, or chess scoresheet, by hand. These moves are usually recorded in a Standard Algebraic Notation (SAN) [2]. This is a standard method for recording and describing the moves based on a coordinate system to uniquely identify each square on the chessboard. This particular move recording standard is the most widely used chess notation in the world, adopted by almost all the chess international organization, including the United States Chess Federation (USCF) and the Fédération Internationale des Échecs (FIDE), which oversee all world-class competitions. Furthermore, most books, magazines, newspapers, and online articles use SAN to report chess games and events. In English-speaking countries, a descriptive notation for chess moves used to be popular until about 1980. While a few players still use the descriptive notation, it is no longer recognized by FIDE [3].

**Figure 1:**
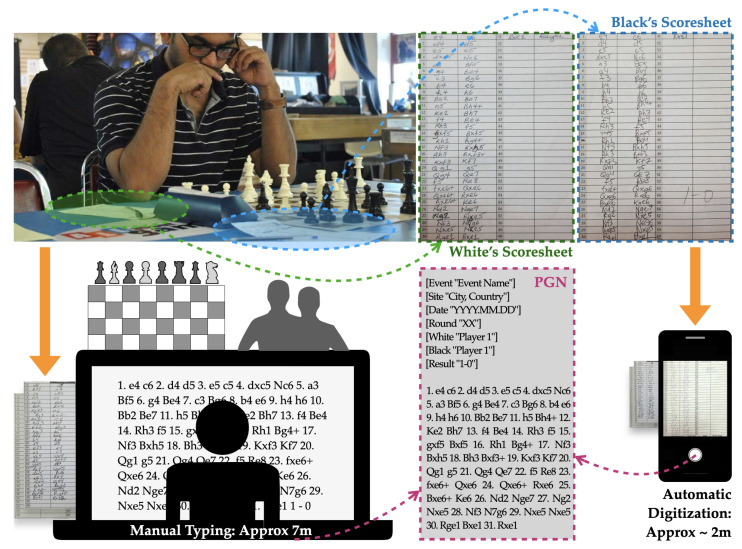
Schematics of an OTB chess event and scoresheet digitization process. **Top** images show the record keeping of moves and collected scoresheets from the players. **Bottom left** shows a potential workflow of manual typing and occasional necessity of a chess board or calling out the players for confusing cases. **Bottom right** shows the proposed automatic workflow of scoresheet digitization which is mostly based on taking pictures with a mobile camera.

The chess scoresheet contains a sequential move history of the entire game along with information about the players, game format, results, and other details like event name, date, board number, etc. They are useful to keep as official game records, and to settle any disputes that may arise, hence their popularity in competitive settings. Moves are never recorded digitally (unless it is an online event) in an attempt to prevent computer assisted cheating, which is a big issue in the world of chess. This scoresheet system, while secure, leaves the event organizers with hundreds of scoresheets which must be manually digitized into a Portable Game Notation (PGN) file. PGN is a standard plain text format (filename extension is .pgn) for recording chess games and other related information, which allows an easy read by humans as well as easy parsing and generation by computer programs [4]. This is the most widely accepted file format for a chess game and is accepted by the majority of the chess programs and engines today. However, the process of converting handwritten scoresheet pages received from the players to PGN files requires extensive time and labor, as organizers must not only type each move, but in the case of a conflict or illegible writing, play out the game on a chess board to infer moves. This is also a stressful and error-prone thing to do, since many conflicts can not easily be fixed without talking with the players. As an alternative to this legacy approach, we propose a deep neural network architecture to perform automatic conversion from pictures of these scoresheets to a PGN text file. In most practical cases, a cellphone camera is an extremely convenient way of collecting data, much more so than a flat-bed scanner. Keeping this in mind, we developed our detection system, from data acquisition to training and testing, to perform digitization on cellphone camera photos. Figure 1 shows an OTB chess event and potential workflows for manual typing and proposed automatic digitization.

This automatic digitization of chess games from scoresheets also provides a benefit for the players. Often, professional and serious chess players like to analyze all of their games later, on their own or with their other chess players or coaches or with a computer engine. This process helps players to track their growth over time, patterns of common mistakes, missed opportunities and scope of improvement in strategies. Since the process of getting a scoresheet and digitizing manually takes a lot of time and effort, many players tend not to go through this crucial self evaluation and reflection stages of their own OTB games. Therefore, an automated process of digitizing a chess game from scoresheet images not only helps the chess event organizers to officially keep their records, but also helps every ambitious chess player who wants to improve their chess skills by studying their own games.

### 1.1. Distinguishing Features of Chess Scoresheets

**Figure 2:**
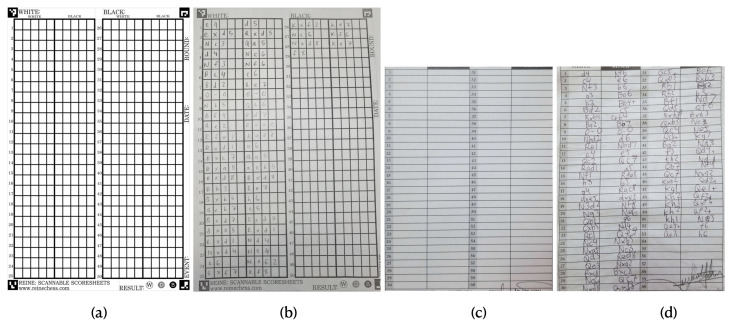
Sample scoresheets from Reine Chess [9] and from an actual chess event: (**a**) Reine blank scoresheet; (**b**) Reine test scoresheet; (**c**) sample standard blank chess scoresheet; and (**d**) sample filled chess scoresheet.

**Figure 3:**
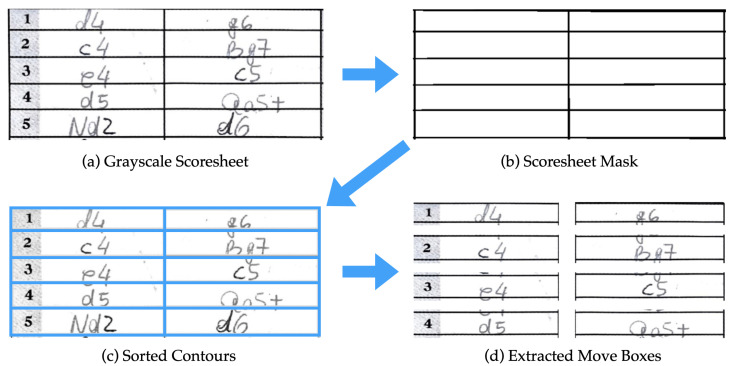
Stages of move-box extraction: (**a**) grayscale image; (**b**) morphological operations to generate a mask; (**c**) contour detection and sorting from the mask; (**d**) sorted contours cropping with top and bottom padding of 15% and 25%.

**Figure 4:**
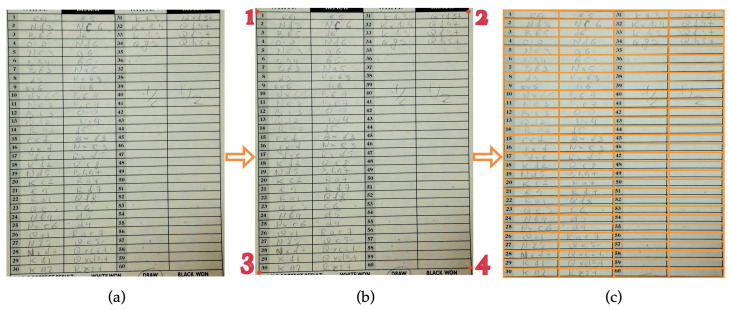
Four-corner-marking is an alternate method for preprocessing which requires a person to highlight the four corners of the move recording table from a chess scoresheet before or after taking a picture: (**a**) a sample scoresheet image; (**b**) marking the four corners of the table with red dots; (**c**) perspective correction and equally dividing the enclosed space to extract individual moves.

In theory, chess moves could be recognized with a standard offline Latin handwriting recognition system, since chess universally uses Latin symbols. This generic approach has its shortcomings. Chess scoresheets offer many distinguishing features that we can leverage to significantly increase move recognition accuracy, including:

- Two scoresheets for each match are available since both players write on their own copy of all the moves played. These copies can be cross referenced for validation.
- Chess moves use a much smaller character set than the entire Latin alphabet, allowing only 31 characters as opposed to the 100+ ASCII (American Standard Code For Information Interchange) characters. Table 1 summarizes the all possible chess move formats allowed in SAN, and Table 2 shows some examples of how they work.
- Traditional post-processing techniques (spell checking, Natural Language Processing or NLP, etc.) do not work. Instead, chess moves can be ruled valid or invalid based on SAN syntax errors or illegality dictated by the game rules.
- Handwritten moves are contained inside well defined bounding boxes (Figure 2, Figure 3 and Figure 4) with some natural degrees of shift; therefore, the process of individual move segmentation is much simpler than in unconstrained handwriting.

Furthermore, the vast majority of chess moves are 2–5 characters long with only a few rare exceptions, and none are longer than 7 characters. These differences between the generic Latin script and handwritten chess moves make it worthwhile to approach this problem separately, while invoking the traditional wisdom of offline handwriting recognition.

### 1.2. Related Works

This paper is an extended version of our formal approach presented in [5], and we found no other academic journal or conference publications for handwritten chess scoresheet recognition at this time of writing. There are some preliminary level works in the form of undergraduate or graduate thesis reports [6,7], but they are not quite well-presented or finished enough to be comparable with our approach. In addition, there are some works on typographical chess move reading from books or magazines (e.g., [8]) which propose ideas for issues like layout fixing and semantic correction, but do not address the problems that can arise from a handwritten scoresheet. Services for digitizing chess scoresheets such as Reine Chess [9] currently exist, but they require games to be recorded on their proprietary scoresheets with very specific formats; they cannot be applied to existing scoresheet formats, and would require tournaments to alter their structure, causing a variety of problems. Figure 2 shows sample scoresheets from Reine Chess and from a typical chess event, which demonstrates the differences and limitations of such scannable solutions in a practical scenario. Scoresheet-specific solutions also offer no solution to retroactively digitize scoresheets and cannot be easily applied to other documents.

Digitizing chess scoresheets is essentially an offline handwriting recognition problem, and there are many different ways this problem can be approached. One approach, known as character spotting, works by finding the locations and classes of each individual component from a word image. This is a powerful technique but is better suited for more complicated scripts [10]. Since the chess moves are recorded using a fraction of the Latin alphabet, a segmentation-free whole word recognition, using tools like Recurrent Neural Networks (RNN) or Hidden Markov Models (HMM), can be considered more suitable for this problem. Our choice for this experiment is a convolutional BiLSTM network. BiLSTM or Bi-directional Long Short Term Memory is a variant of a Recurrent Neural Network (RNN), which has been proven to be extremely powerful in offline recognition in recent years. For example, Bruel et al. achieved a 0.6% Character Error Rate (CER) using a BiLSTM network for printed Latin text [11]. Shkarupa et al. achieved 78% word-level accuracy in classifying unconstrained Latin handwriting on the KNMP Chronicon Boemorum and Stanford CCCC datasets [12]. Dutta et al. were able to achieve a 12.61% Word Error Rate (WER) when recognizing unconstrained handwriting on the IAM dataset [13]. Ingle et al. proposed a line recognition based on neural networks without recurrent connections and achieved a comparable training accuracy with LSTM-based models while allowing better parallelism in training and inference [14]. They also presented methods for building large datasets for handwriting text recognition models. In contrast, Chammas and Mokbel used an RNN to recognize historical text documents demonstrating how to work with smaller datasets [15]. Sudholt et al. presented a Convolutional Neural Network (CNN), called Pyramidal Histogram of Characters or PHOCNet, to deploy a query based word spotting algorithm from handwritten Latin documents [16]. Scheidl et al. demonstrated the benefit of training an RNN with the Connectionist Temporal Classification (CTC) loss function using the IAM and Bentham HTR datasets [17].

Pattern recognition systems are never error-free and often researchers propose systems with human intervention for assistance with certain predictions [18,19]. We also used such a strategy that we call semi-autonomous post-processing, which looks for manual help for unresolved cases. Different segmented text recognition approaches for problems like bank check recognition, signature verification, tabular or form based text identification are also relevant to our chess move recognition problem. One notable demonstration for recognizing segmented handwritten text was presented by Su et al. using two RNN classifiers with Histogram of Oriented Gradient (HOG) and traditional geometric features to obtain 93.32% accuracy [20].

### 1.3. Overview of the Presented Approach

This paper presents an end-to end system for offline chess scoresheet recognition with a convolutional BiLSTM neural network as an alternative to the existing inefficient method of manual digitization. To accomplish this, we pretrain a deep neural network on an existing Latin handwriting dataset, IAM [21] and later fine-tune our model with redefined classes and network size adjustments. Each handwritten move is extracted from its scoresheet during pre-processing and passed through the network to generate a prediction. We used post-processing algorithms to restrict the output and improve accuracy, since valid chess moves allow only a limited set of letters, numbers, and symbols (Table 1 and Table 2) in specific positions (e.g., moves cannot start with a number, cannot end with a character, etc.). We also utilized the fact that all moves are not legal from a certain board position; therefore, a valid notation can still be an illegal move and can be detected if we can keep track of the pieces on the board. For example, a piece can not move to a square which is already occupied with a piece of the same color, rooks can not move diagonally and a light square Bishop can not land on dark square. There are plenty of chess engines available which already can tell if a move is legal or not. We used one of them, Stockfish 14.1 [22], in our post-processing. Leveraging the fact that always two scoresheets are available for a game, we proposed two post-processing mechanisms: an autonomous post-processing which outputs a final prediction for the text box after cross-checking with the game’s second scoresheet; and a semi-autonomous post-processing that increases the recognition accuracy to a near human-level by requesting user input for difficult handwriting samples, invalid entries and unsettling conflict cases. Although the semi-autonomous correction is an interrupting process, it can also save hundreds of hours for event organizers and drastically reduce human labor required for digitizing chess games.

We made the Handwritten Chess Scoresheet (HCS) dataset, which we used for our experiments, openly accessible to encourage further research and development [23]. This dataset contains scoresheets from actual chess events digitized with a cellphone camera and tagged with associated ground truths. This also has some additional data containing only less frequent chess moves to allow a network to be trained more uniformly. The HCS dataset is currently the only publicly available chess scoresheet dataset. As this is an extension of our original work presented in [5], we present the details of our entire approach highlighting the changes we made and improvements we achieved since our first attempt.

## 2. Offline Chess Scoresheet Recognition

### 2.1. Preprocessing

A chess scoresheet has predefined boxes where players enter moves during a game. While there are different styles of scoresheets, every one that we encountered ordered moves in essentially the same way: four columns, each representing 30 moves, with columns and rows separated by solid grid lines. To train our network to recognize these moves, we developed an algorithm that isolates each move based on those grid lines. First, we convert our RGB images to gray-scale, and then to binary images using a threshold obtained using Otsu’s method [24]. Then, we use two long, thin kernels (one horizontal and one vertical) with sizes relative to input image dimensions, and morphological operations (erosion followed by dilation) with those kernels to generate an image containing only grid lines. With this simplified image, we use a border following algorithm [25] to generate a hierarchical tree of contours. Each contour is compressed into four points, storing only corners of each quadrilateral. Any contour which is significantly larger or smaller than the size of a single move-box (again, calculated relative to the total image size) can be ignored. The final contours are sorted based on their positions relative to one another, and each is labeled by game, move, and player. Finally, we apply a perspective correction to restore the original rectangular shape of the move-boxes and crop each of them with a padding on the top and bottom of 15% and 25%, respectively, since written moves overflow the bottom of their box more often and more severely than the top. This process is displayed in Figure 3. We did not pad box sides because chess moves are short, and the players rarely need to cross the side boundaries. This method of pre-processing is nearly agnostic to scoresheet style and will work with any scoresheet style, which includes 4-columns and solid grid lines.

With professional settings like chess events, often achieving the highest possible precision is an absolute priority even if it has to come with a little sacrifice in convenience. Keeping that in mind, we also developed an alternate four-corner-marking method for chess move extraction which further minimizes the chances of performance degradation due to a preprocessing error. With this method, a person needs to mark the four corners of the moves recording table on a chess scoresheet with red circles. Figure 4 shows the workflow of this alternate method. Marking can be done before or after taking the picture. While this additional process makes the overall scoresheet digitization process a tad slower, it also increases the recognition accuracy a little more as discussed in Section 4. This is because, after getting the coordinates of the four corners of the table, the segmentation is done by perspective correction and then equally dividing the space. This is an optional process we developed to be used in order to get the best possible recognition performance out of our system. Although for our dataset, the four-corner-marking method did not improve the results drastically, it might be more useful in other formats of scoresheets like those with graphics and text in many different places or poorly captured sample images where the lines and borders are not well pronounced. This method also provides an option for chess clubs or organizations to deploy a four-corner-marking on their template scoresheet to allow a better and faster corner detection, therefore facilitate a more reliable automatic chess move digitization process.

### 2.2. BiLSTM Neural Network Architecture

We used a BiLSTM convolutional neural network as presented in [5] for recognizing chess moves from the extracted images. This architecture has been previously used by Shi et al. for image based sequence recognition [26]. The network includes 10 layers, separated into three functional groups. The first seven layers are convolutional and take in the gray-scale image of a single move-box, scaled to a resolution of 64×256 pixels. These layers act as feature extractors and convert image data into a feature map. This feature map removes unnecessary information and creates a sequence input for the recurrent layers. The recurrent model itself consists of two BiLSTM layers, which take the feature map and generate a sequence output to a final dense network. The BiLSTM neural network is a modification of the traditional RNN which takes advantage of hidden memory units to better process sequence data. The final dense network converts the LSTM sequence output into a loss matrix. Afterwards, a Connectionist Temporal Classification (CTC) loss function [27] is applied, which converts this matrix into a string. The CTC loss function and decoder better estimate the network loss than more general loss functions and allows for simple repeat errors common to the LSTM layers to be corrected. This loss matrix encodes a string as a vector of one-hot encoded character vectors, so the final matrix has dimensions of the number of allowed characters by maximum output length (plus a small padding on output length for blank characters).

**Figure 5:**
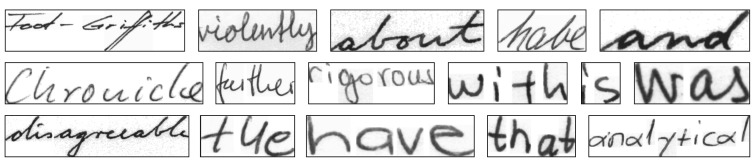
Sample images from the IAM dataset [21] used for pretraining.

First, we pretrained our network with the IAM dataset [21], one of the most used collections of unconstrained handwritten English sentences. Figure 5 shows several sample images from the IAM dataset. Approximately 86,000 words of this dataset were used to pretrain our network so that it could learn the overall Latin script and later be modified for chess move recognition. We used the Adam optimizer with a learning rate of 0.0005 for 10 epochs for this pretraining. Adam is a first-order gradient-based optimization of stochastic objective functions which is computationally efficient and considered well suited for similar problems [28]. In addition, this process of transfer learning is a widely used technique which can achieve faster and better network training and is especially useful when working with a small dataset. While pretraining, the network uses two BiLSTM layers with 256 hidden units and a dense network which outputs a loss matrix of 81×31. These dimensions allow pretraining on the IAM dataset which has a total of 80 allowed character classes (entire Latin alphabet, numerals and frequently used symbols) and a maximum word length of 27.

**Figure 6:**
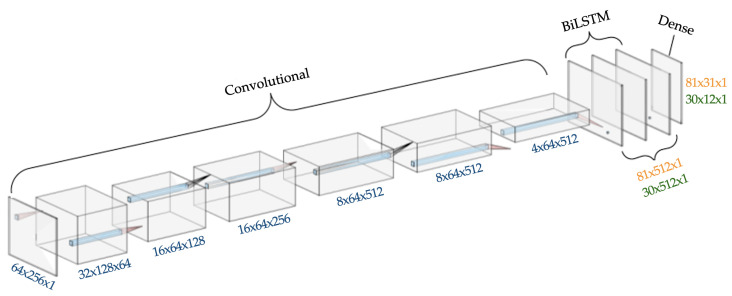
Layer graph of the BiLSTM network. Orange and green values show the dimensions for the pretraining network and our chess move recognition network, respectively. Pooling, batch normalization, and reshaping layers are not shown.

Afterwards, we restructured this pretrained network for chess move recognition. The SAN format uses 30 allowed characters with a maximum possible move length being 7. To match with the SAN system, we reduced the BiLSTM layers from 256 hidden units to 64, as well as re-tuned the final dense layer to output a loss matrix of 30×12 instead of 81×31. We trained the network for 50 epochs with the similar settings as our pretraining process. Figure 6 shows the network with both the pretraining and fine-tuning structures. The maximum length from the output is slightly overestimated, allowing the network to have additional characters to compensate for CTC loss ‘blank’ characters and double spotting of a single character (which frequently occurs with LSTM layers). Since the SAN system does not include any valid chess moves with adjacent repetition, these extra characters are completely removed during the decoding phase. The whole experiment was done using open source API Keras with TensorFlow 2 in the backend.

The choice of BiLSTM network is neither the key of our approach nor the only option to develop such a handwritten chess move recognition framework. Many other Latin offline recognition frameworks, such as HMM or Gated Recurrent Unit (GRU) based architectures should work equivalently well if the output classes are reduced to allow only the valid chess move symbols. The aim of this research was not to test all possible tools and find the best fit for this experiment, rather to have a good starting point and fine-tune the recognition performance by applying chess-specific rules and attributes. The network of our choice, i.e., the convolutional BiLSTM is considered one of the most powerful tools in offline handwriting recognition. This is a variant of Recurrent Neural Network (RNN) which is not susceptible to vanishing gradients. The LSTM works with sequence input and output data, allowing for variable length moves, which is crucial for a chess game (for example, both ‘e4’ and ‘Qh4xe1+’ are valid algebraic notation moves). In addition, an LSTM-based network does not require labeled bounding boxes around individual characters, which involves a much more intensive ground-truth tagging process. The network’s consideration of the entire input image and not just a single character allows it to take advantage of context clues, which are often extremely important when decoding chess moves. If the network can gain a general understanding of the syntax of algebraic notation (e.g. that the first characters of a move are often in the form ‘Piece’ ‘a–h’ ‘1–8’, e.g., Nf3), then it can make much more accurate inferences about the ground truth of an ambiguous character. The Long Short-Term Memory (LSTM) cells retain information seen earlier in the sequence more effectively, and the bidirectional LSTM or BiLSTM structure allows the network to make predictions using information in both the positive and negative time directions. This bidirectional movement makes inference easier, allowing the network to leverage context from the entire input as it reads. The GRU networks can function as lighter versions of LSTMs, since they behave similarly but use fewer trainable parameters; therefore, they are relatively faster to train [29]. However, we preferred the BiLSTM network because, in most cases, LSTMs outperforms GRUs in terms of recognition accuracy, which is a key concern for a framework to be reliable in practical use.

### 2.3. Post-Processing

Given the availability of two unique scoresheets for each game (one written by each player), the fixed structure of the algebraic notation for every chess move, and the possibility to check whether a move is legal by assessing the current board position, we developed two simple post-processing strategies:

#### 2.3.1. Autonomous System

The autonomous system relies on cross-checking between white and black scoresheets and spotting invalid notations to improve accuracy. Here, invalid notation refers to a syntactic error or an illegal move with respect to game rules. Anything beyond the scope of acceptable SAN notation (Table 1) is considered as a syntactic error. To spot an illegal move, we keep the record of the current board position as the system recognizes each move, and then use an open source chess engine, Stockfish 14.1 [22], to check whether the predicted move is possible or not. The system compares two predictions for each move along with their confidence values. The confidence value is calculated as the exponential of the negative CTC loss between the raw network output and its decoded string [30]. The system then makes the following decisions:

- If the predictions agree, the system accepts that prediction regardless of their confidence values.
- In case of a prediction conflict where both of the moves are valid, the prediction with higher confidence score is accepted.
- In case of a prediction conflict where both of the moves are invalid, the prediction with higher confidence score is accepted.
- In case of a prediction conflict where one of the moves is invalid and one is valid, the valid prediction is accepted regardless of confidence values.

**Figure 7:**
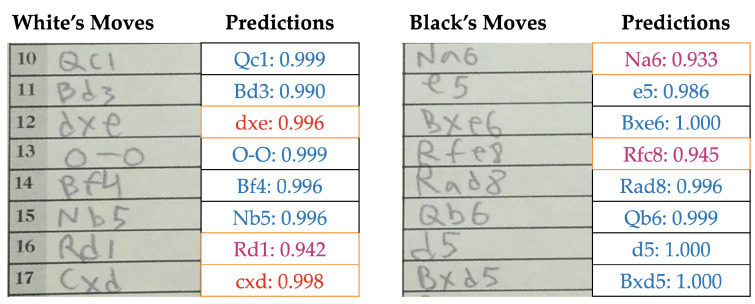
The network output of a scoresheet portion as presented to the user. Low confidence predictions (<95%) such as ‘Na6’ are presented in magenta, and invalid or illegal moves such as ‘dxe’ are presented in red for quick lookup and correction.

For example, if the network prediction is ‘NG4’ (invalid) for one sheet and ‘Ng4’ (valid) for the other, the final prediction becomes ‘Ng4’, regardless of their confidence scores. Alternatively, if both moves were valid (e.g., ‘Ng4’ and ‘Ne4’) or both invalid (e.g., ‘NG4’ and ‘NG8’), the system then accepts the one with the higher confidence regardless of their validity. After all predictions of a game, both scoresheets are presented to the user with confident predictions labeled in blue, lower-confidence predictions highlighted in pink and invalid or illegal move predictions highlighted in red as shown in Figure 7. We chose a confidence threshold of 95% in this autonomous approach. This allows the user to take a brief look after the network generated results and spot obvious issues.

#### 2.3.2. Semi-Autonomous System

To increase accuracy even further, we can leverage the user to provide input on certain moves where the predictions are likely to be wrong. This allows significant improvements to accuracy with minimal interruption to the user. As with autonomous post-processing, we take in two sets of predictions with their respective confidence values, one for the scoresheet written by each player. We classify each prediction in each set as *confident* if it meets a confidence threshold of 90%, *valid* if a legal move based on the board position is syntactically correct. In the following cases, the algorithm makes a decision without interruption:

- If both predictions are the same and *valid*, and if at least one of them is *confident*, the prediction gets accepted.
- If predictions are different, but only one of them is *valid* and *confident*, that prediction gets accepted.

If neither of these two cases applies, the system then seeks a manual help by interrupting the recognition process. Things that can cause this user interruption are as follows:

- Zero Match: When none of the predictions are *valid* and *confident* at the same time.
- Weak Match: When the predictions are *valid* and same, but none of them is *confident*.
- Strong Conflict: When the predictions are different, but both are *valid* and *confident*.
- Weak Conflict: When the predictions are *valid* but different and none of them is *confident*.

**Figure 8:**
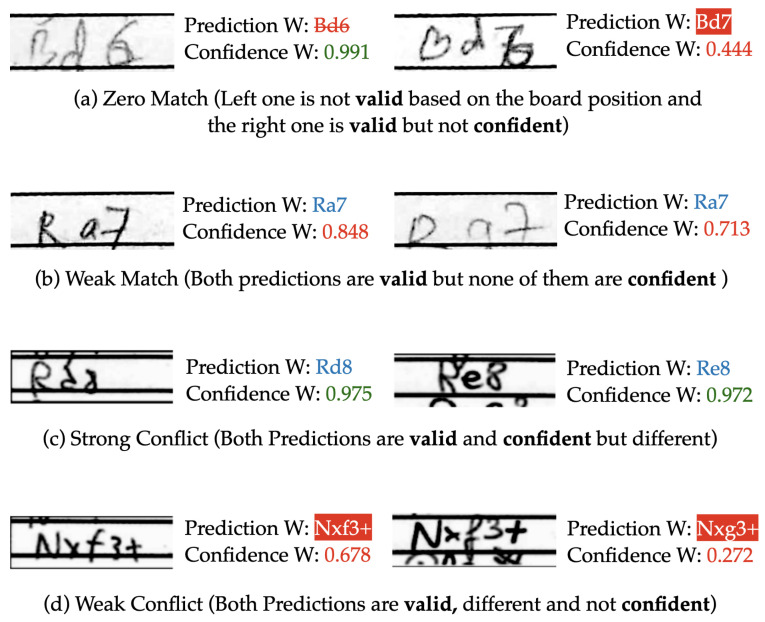
Example cases where the user is interrupted for a manual entry in semi-autonomous post processing system: (**a**) zero match; (**b**) weak match; (**c**) strong conflict; (**d**) weak conflict.

**Figure 9:**
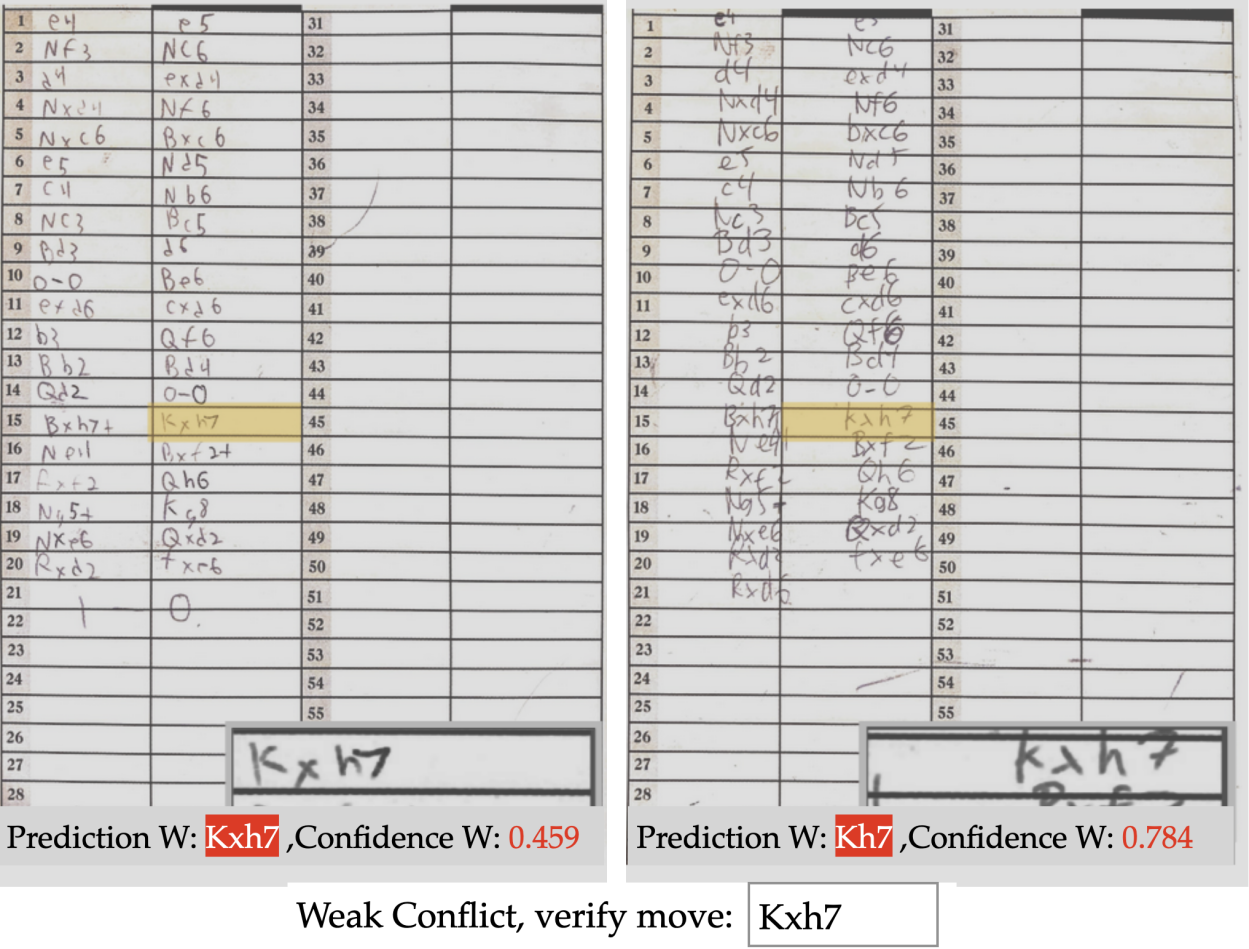
The semi-autonomous user interface, displaying both scoresheets with the pertinent moves highlighted, along with a zoomed view of the moves and the network’s guess for each.

Figure 8 shows examples of all four possible cases where the semi-autonomous system invokes for a human intervention. For the Zero Match case, the move ‘Bd6’ was not considered *valid* based on the board position, and the move ‘Bd7’ was *valid* but not *confident*. For the Weak Match example, both predictions were ‘Ra7’ and that was a *valid* move. However, none of those predictions were *confident*. The Strong Conflict example involves two *confident* and *valid* but mismatched predictions of ‘Rd8’ and ‘Re8’. The *weak* conflict example shows predictions of ‘Nxf3+’ and ‘Nxg3+’, each with low confidence values. Although it is possible to have a chess engine assisted suggestion based on the predictions for all four of these cases, it is much simpler and more reliable to prompt for a human intervention to resolve the issue. Therefore, while sacrificing a little convenience in the automation process, our semi-autonomous workflow ensures a much better PGN conversion. When this interruption occurs, we display the full scoresheets along with the network’s predictions, and ask the user to input the correct move. The user interface of a sample case of Weak Conflict is shown in Figure 9.

### 2.4. Data Augmentation

Data augmentation is one of the most widely used techniques in machine learning. This is a way to increase the amount of data by adding slightly altered versions of already existing data. It works as a regularizer and resists overfitting when training a neural network model. Since our current HCS dataset [23] is certainly on the smaller side for training a deep neural network, it was necessary to increase the training set size by data augmentation. By applying meaningful image transformations to each move-box image, it is possible to simulate a larger dataset, which almost always ensures a more generally trained network. We generated 10 augmented images for each sample using one of the following transformations and range randomly:

- Rotation (between −10° to 10°);
- Horizontal scaling (between −20% to 20%); and
- Horizontal shear (between −15° to 15°).

**Figure 10:**
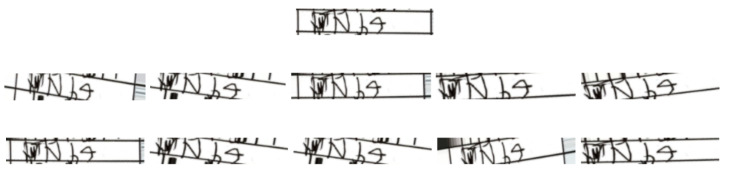
Original image (on top) and ten augmented images. Ground truth: ‘Nb4’.

Figure 10 shows a few samples of these augmented images. There are many other kinds of data augmentation techniques available that could have been potentially used with our system, but we kept this process simple with just the basic techniques.

## 3. The Handwritten Chess Scoresheet (HCS) Dataset

**Figure 11:**
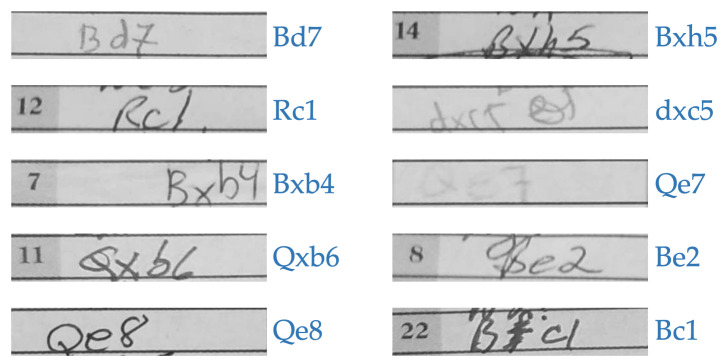
Clear samples (**left**) and messy samples (**right**) from the HCS dataset [23]. Original moves are annotated beside the samples in blue.

There were no publicly available chess scoresheet image datasets that we could use when we first presented this approach in [5]. Therefore, we developed our own, the HCS dataset [23], to train and test our network. This dataset consists of 158 total games, 53 of which had both copies from the players with white and black pieces and the remaining 105 with just one copy, making a total of 211 pages of chess scoresheet images. All the images were digitized using a standard cellphone camera in natural lighting conditions. These images are tightly cropped, and a standard corner detection based transformation is applied to eliminate perspective distortion. The headers and footers were also cropped out from each image in order to maintain player anonymity. The scoresheets were collected from actual chess events; they were not artificially prepared by volunteers. Despite posing challenges for training, identifying handwriting “in the wild” has more generality than identifying text from artificial, pristine images. The data are therefore very diverse, consisting of many different handwriting and scoresheet styles, varieties in ink colors, natural occurrences of crossed-off, rewritten and out-of-the box samples, a few of which are shown in Figure 11.

**Figure 12:**
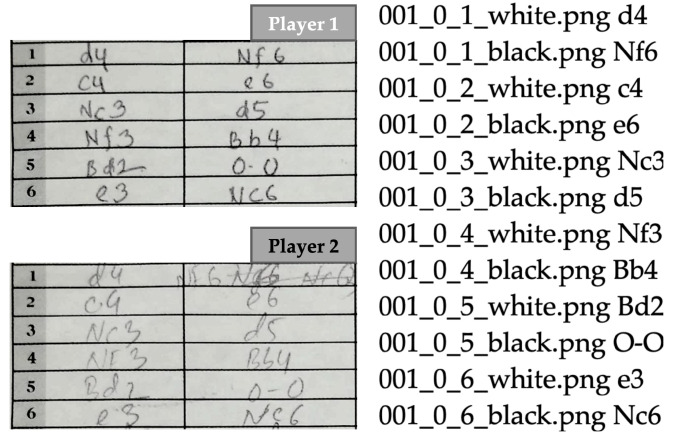
Cropped samples of raw images (**left**) and associated ground truth labels (**right**) from the HCS dataset [23].

Each scoresheet in the dataset contains 120 text boxes (60 boxes for each player). However, many of these boxes are empty since most chess matches last for far fewer than 60 moves. Omitting the empty boxes, there are approximately 13,810 handwritten chess moves currently in our dataset, with an average of approximately 33 moves (66 for each player) per game. Some of these scoresheets came with a photocopied or carbon copied version as well. These were included in the dataset since such variations of images provide a natural form of data augmentation which can be useful for the training process. We manually created the ground truth version of each game and stored it in a text file. A sample is shown in Figure 12. Both the images and the ground truth text files are stored with a naming convention given by:

[game#]_[page#]_[move#]_[white/black].png/txt

Furthermore, we created an additional dataset to improve the overall balance of our training data. One of the major problems with our first attempt as reported in [5] was caused by moves that occur rarely. For example, short castles (written as ‘O-O’) are way more common than long castles (written as ‘O-O-O’). This also reflects our dataset as we have around 10 times more samples of castles short than long. Since the network was not well trained with enough data to recognize the long castles, it consistently predicted those as short castles too, which greatly affected the recognition accuracy in our earlier attempt. Other moves like pawn promotions (e.g., ‘f8 = R’) or disambiguating moves (e.g., ‘Nbd7’ or ‘R1f7’ ) are also very rare, and we did not have enough samples in our dataset that is required for a robust training. Therefore, we opted to craft a small dataset of less frequent moves to mitigate this problem. The images of this dataset were not collected from actual games, rather artificially created with the help of volunteer chess players. This contains:

- 20 samples of long castles or ‘O-O-O’.
- 20 samples’ pawn promotions from a random file (between ‘a’ and ‘h’) to random promotion pieces (‘Q’, ‘R’, ‘B’ or ‘N’), a few with random events like capture, check, or mate.
- 20 samples’ random disambiguation moves, 10 from disambiguation files and 10 from disambiguation ranks a few with random events like capture, check, or mate.

**Figure 13:**
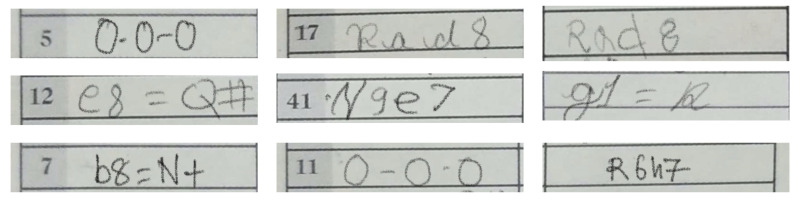
Samples from the less frequent moves dataset. These images are not taken from real games, rather created artificially with the help of volunteers. This is also a part of the HCS dataset.

A set of sample images from this less frequent moves dataset is shown in Figure 13. We used the same data augmentation techniques described earlier with this additional dataset as well.

To summarize, this release of the HCS dataset contains:

- 211 images of scoresheets from 158 unique games collected from actual chess events. Only 53 of these games come with both players’ scoresheet,
- 60 images of less frequent moves dataset artificially created with the help of volunteer chess players,
- 8 samples of empty/blank scoresheet images and
- 25,327 extracted move box images which include 13,810 of written moves and 11,517 of unused blank boxes from the scoresheets.

The HCS dataset is public and free to use for researchers who want to work with similar problems [23].

## 4. Training and Results

We used the following set of data for training our BiLSTM network:

- 161 scoresheet images from our HCS dataset. This includes 28 games where both players’ scoresheets were available and 105 games with single scoresheets. There are 9370 raw move images which with our 10:1 data augmentation scheme expanded into 93,700 training images.
- 60 additional images of less frequent chess moves from the HCS dataset (described in Section 2.4 which after augmentation expanded into 600 additional training images.
- 15 annotated scoresheets or 1160 move images collected from web scraping which after the same augmentation scheme expanded into 11,600 additional training images.

This entire combination gave us a total of 105,900 move images which is a decently sized dataset to obtain a robust training. These data are separated into batches of 32, and trained with a learning rate of 0.0005 using the Adam optimizer for 50 epochs. For testing, we used 25 games or 50 scoresheets, which translates into 1905 testing image pairs or 3810 total move images. This is roughly 15% of our HCS dataset. This test set was composed of data from writers/players unseen by the network during training. Not all of the games in our dataset have unique white and black player copies, which is fine for the network training with our approach. However, since our post-processing pipeline uses a comparison framework from two copies of the same game, the test set is carefully chosen with the games where both white and black player scoresheets are available, which also means that the user needs to submit both scoresheets for a game in order to use our post-processing schemes.

**Figure 14:**
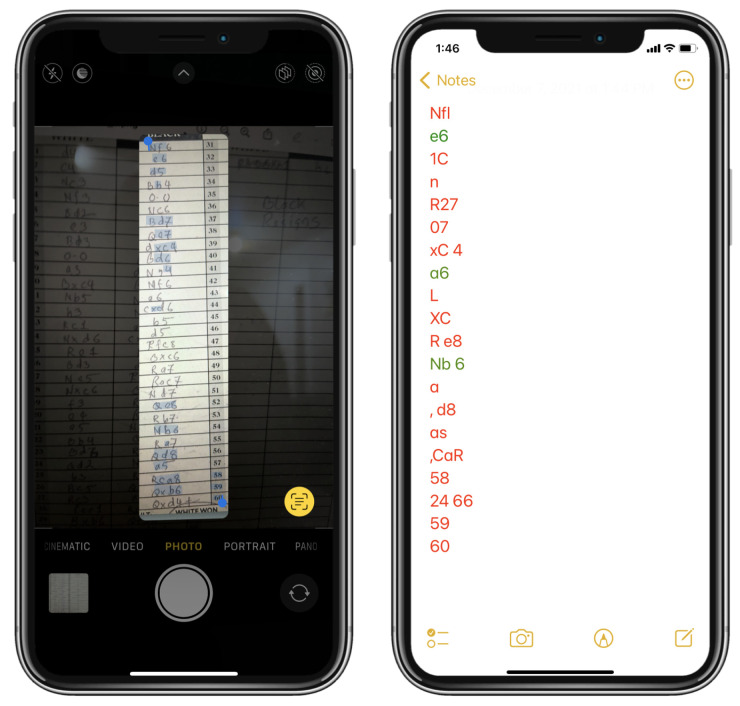
Demonstration of the Live Text feature in iOS 15 with an iPhone [31]. The left image shows the Live Text detection on a column of a test scoresheet, and the right image shows the predicted outputs. The correct predictions are colored green and the incorrect predictions in red.

Table 3 presents the performance of our system with different pre and post processing conditions. We used the Live Text as introduced in iOS 15 [31] and macOS Monterey 12 [32] by Apple Inc. (California, USA) to its supported devices like iPhones, iPads, Mac computers, etc. to compare our raw move recognition accuracy against an off-the-shelf solution to this problem. The Live Text feature attempts to spot and recognize texts from an image, and generally it works great with handwriting. The move recognition accuracy we obtained with Live Text on a smaller test set is less than 20% compared to the 87.8% of raw recognition accuracy we obtained without any pre or post processing. A sample showing how Live Text struggles to recognize the moves is shown in Figure 14. This goes to show why a generic offline handwriting recognition solution might not be a viable option for a specialized problem like this.

As can be seen from Table 3, the effect of the four-corner-marking preprocessing over our default text box extraction method is fairly small (around 1%) but is consistent across every test scenario. The four-corner-marking approach comes with a cost of convenience, since the user needs to manually mark the corners of a scoresheet table (Figure 4. Therefore, the default text box extraction method might be more desirable for most practical cases. However, in situations where the absolute best performance out of the system is needed, or maybe with specific types scoresheets where the default text extraction process is not functioning well, the four-corner-marking is a more reliable option for our system.

The autonomous post-processing with default text box extraction is an uninterrupted workflow, which gave us a move recognition accuracy of 94.9%. This translates into less than four error cases for a game of average length. This already competes with the legacy approach of pure manual entry with casual human error, especially when recording a lot of games at a stretch. Furthermore, we present the complete scoresheet along with the predictions and their corresponding confidence values. During this presentation, if a move is invalid or illegal, we mark it with red and if the move is legal but below a confidence threshold of 95%, we mark it with magenta. This allows the user to quickly spot, check and correct for any prediction errors or inconsistencies. Figure 7 shows an example prediction output of our autonomous pipeline.

The most drastic improvement is found with the semi-autonomous system with a recognition accuracy of 98.4% (99.1% with four-corner-marking). This is around 11% gain in accuracy, while only requesting user input on 4% of the moves. This means, for a 30 move game (30×2 total moves from both players), a user will be prompted for manual entry 2–3 times on average. In our case, many scoresheets went without any interruption, while, for a few faded and quite illegible scoresheets, it was invoked frequently. The best error rate of 0.9% we obtained out of the system using the four-corner-method combined with semi-autonomous post-processing. This is arguably much better than what we expect from the legacy approach of manual entry. Furthermore, many of these errors are caused when players accidentally write different moves, and one of those moves is above the confidence threshold while the other is not. This means that the user is not prompted for the move (considering both of the moves are valid and legal), and whichever has a higher confidence value is selected. This selection, whether correct or not, is currently counted as an error since the selected move contradicts one of the two samples we have from the players. This error is common, so our true accuracy may be slightly higher than the measured accuracy of 99.1%. Since our current ground truth values are based on the handwriting itself, and not what was played in the game, we may be outputting a prediction which is correct to the game, but not to the handwriting of one scoresheet.

Errors from both autonomous and semi-autonomous systems are primarily caused by incorrect prediction, when the network does not accurately recognize an individual move. These errors generally fall into one of three categories: rare move structures (~20%), illegible writing (~50%), or information lost due to cropping (~30%). Rare or less frequent moves such as pawn promotions, moves involving disambiguation files/ranks, or long castles were a major problem in our first attempt [5]. While easily recognizable to a human, our real event dataset includes very few (or sometimes no) examples of these move structures, so the network tended towards a simpler and shorter prediction. With the introduction of the less frequent moves dataset as described in Section 3, the issues with the rare structured moves have been heavily mitigated. Ideally, a larger dataset should reduce this type of error even more. Messy, illegible writing which includes crossed-out or cramped letters (examples shown in Figure 11) often causes single move errors; however, most of these are solved with our post-processing algorithm. Both the autonomous and semi-autonomous algorithms cross-check both player’s scoresheet, one of which is likely to be written in a better way. Finally, many players do not write a move entirely within its move-box, and this missing information also causes prediction errors.

Along with reporting the excellent performance, we also wanted to quantify the amount of time our different approaches take compared with the time it takes for pure manual entry. In this regard, we collected data by talking with a number of chess event organizers and by involving a number of volunteers to digitize scoresheets using our framework with different pre- and post-processing approaches. The summary of this timing research is presented in Table 4. Although this is a very small scale research, it should reflect the actual time requirements with a reasonable degree of error. As can be seen, our worst workflow still is twice as fast as best possible cases of manual entry when there are no conflicts or issues with the scoresheets. In addition, these numbers add up pretty quickly for a medium to large tournament. For a chess event with 31 games (e.g., FIDE World Cup 2021), even our most time-consuming workflow can save more than two-and-half hours of manual labor while giving a 99.1% move recognition accuracy. It is tough to estimate how many OTB chess events happen everyday around the globe, but, for sure, that number is a huge one. This implies that our framework or frameworks like this can have a massive impact in the chess world events, let alone the convenience it provides to a player for his/her own growth.

Since, we could not find any similar work reported for handwritten chess scoresheet recognition, and off-the-shelf solutions like Apple’s Live Text are not useful for this problem, we can only compare our work to our former attempt, as presented in [5]. For the autonomous post-processing, our character recognition accuracy and move recognition accuracy went up by 2.7% and 5.5% respectively. For the semi-autonomous post-processing, we improved our character recognition accuracy and move recognition accuracy by 1.5% and 1.9%, respectively, while the interruption rate went down by 3%. This improvement is caused by a number of things. Firstly, we used more than twice the amount of data. We utilized the Stockfish chess engine to further identify possible errors in detection. Lastly, we crafted an additional dataset for rare occurrence moves which balanced our dataset a little more than our previously used training set. Obviously, the system will become more robust with more data, but there is still room for improvement in post processing. Currently, our evaluation metric is based on what the players wrote, but not what the actual game is. With this approach, if a player mistakenly writes a valid move which is not what he/she played, the move might not be immediately considered as an illegal move. Rather, at a later stage of the game, a contradiction might occur causing an actual true move to be labeled illegal. This problem can be resolved if we can keep track of all probable paths of the game, and at the end we would present the entire game path which causes the least amount of contradictions with what the players wrote in their scoresheets.

## 5. Conclusions

Although the core of this approach of scoresheet digitization was first presented by us in [5], we have made a substantial amount of progress since then. We upgraded our dataset and training process, introduced a more reliable preprocessing technique and improved our post-processing pipeline by utilizing a chess engine. Starting with this relatively small HCS dataset, we were able to achieve an accuracy of almost 95% with no human interaction needed other than just to take the photos with his/her cell phone camera. This autonomous approach almost instantly presents the resultant scoresheet to the user with highlights for the low confidence predictions and possible illegal or invalid moves. This also allows the user to quickly correct a few moves if needed. Our semi-autonomous approach increased this accuracy to around 99% with an average of less than three interrupts per game needed for human intervention. Although this accuracy is potentially way better than actual human level performance in most practical scenarios, even a 1% error rate can cause big issues in some sensitive cases. This small gap from a perfect result could be further reduced by getting more data for training and adding more samples of rare occurrence moves whether synthetically or organically. Along with that, a more sophisticated post-processing system could be deployed which not only makes predictions move by move, but presents an entire game with least edit distance required from the series of predictions. With tools from graph theory, it could be possible to determine the fewest number of alterations required in order to have a top-to-bottom valid game—this could take the system accuracy even closer to a 100% accurate system. Another possibility is to utilize the most played moves in a certain position in the post-processing, which could be especially useful in common chess openings. Thanks to the rapid growth in online chess, lots of open source databases are available to get this information. In order to encourage further research and development in this area, we offer our HCS dataset to the public [23]. We also have a future plan to make this framework openly accessible to the chess community.

The applications of deep learning have been assisting us in countless different ways, and yet almost no meaningful attempt has been made to resolve this seemingly obvious problem of chess scoresheet digitization. The chess world is a gigantic community and the number of events and activities is always growing. A handwriting recognition framework can be particularly useful with chess since players are bound to record moves by hand in order to prevent computer aided cheating. Although the process of digital record keeping with human labor has never been considered as a problem, we do not have to keep the legacy approach when and if the technology allows us to improve. Here, we present an attempt to save thousands of man-hours currently being spent by event organizers to transform chess scoresheets into standard PGN files. While saving a substantial amount of manpower, our framework also makes the record-keeping process extremely convenient and arguably more reliable. Chess scoresheet digitization has a variety of uses from post-game engine analysis to efficient game-publishing, and in most professional and serious events, it is mandatory to keep these records. Therefore, we firmly believe that our presented approach can have a big influence, and potentially revolutionize the chess event management system practiced today.

## Figures and Tables

**Table 1:** Standard Algebraic Notation (SAN) for chess moves. Actual notations are shown in orange and the corresponding descriptions are shown in black. Short and long castles (not included in the table) are denoted by O-O and O-O-O respectively [5].

Piece	Disambiguating File/Rank	If Capture	Destination File	Destination Rank	If Promotion	If Check or Mate
Pawn ∼ (none) King ∼ K Queen ∼ Q Rook ∼ R Bishop ∼ B Knight ∼ N	Either from a–h or from 1–8 for disambiguating File or Rank respectively	X	One choice from a–h	One choice from 1–8	= followed by the Piece Q, R, B or N	Check ∼ + Mate ∼ #

**Table 2:** Example chess moves with their descriptions and how they are interpreted in SAN format. Dis. or disambiguation file or rank is used only when multiple pieces can move to the same destination square.

Sample Move	Move Description	Piece Name	Dis. File	Dis. Rank	Captures on	Dest. Square	Promotion Piece	Check or Mate
Bf3	*Bishop to f3*	B	∼	∼	∼	f3	∼	∼
R2f4	*Rank 2 Rook to f4*	R	∼	2	∼	f4	∼	∼
Bxd5	*Bishop* *captures on d5*	B	∼	∼	x	d5	∼	∼
Ref8	*e-file Rook to f8*	R	e	∼	∼	f8	∼	∼
Qh4f2	*h4 Queen to f2*	Q	h	4	∼	f2	∼	∼
e8=R	*Pawn to e8,* *promotes to Rook*	∼	∼	∼	∼	e8	=R	∼
e5	*Pawn to e5*	∼	∼	∼	∼	e5	∼	∼
Qxf7#	*Queen captures on* *f7 and Checkmate*	Q	∼	∼	x	f7	∼	#
Bxc3+	*Bishop captures on c3* *and Check*	B	∼	∼	x	c3	∼	+
dxe5	*d-file Pawn* *captures on e5*	∼	d	∼	x	e5	∼	∼
O-O	*Short Castle*	∼	∼	∼	∼	∼	∼	∼
O-O-O	*Long Castle*	∼	∼	∼	∼	∼	∼	∼

**Table 3:** Character Recognition Accuracy (CRA), Move Recognition Accuracy (MRA), and Interruption Rate are for various levels of pre and post-processing.

Preprocessing	Post-Processing	Interruption Rate	CRA	MRA
Text Box Extraction	None	N. A.	94.3%	87.8%
Four-Corner-Marking	None	N. A.	94.7%	89%
Text Box Extraction	Autonomous	N. A.	97.7%	94.9%
Four-Corner-Marking	Autonomous	N. A.	98.2%	95.6%
Text Box Extraction	Semi-Autonomous	4%	99.3%	98.4%
Four-Corner-Marking	Semi-Autonomous	4%	99.6%	99.1%

**Table 4:** Timing estimation for digitization of handwritten chess scoresheets with different approaches and scenarios. Numbers presented are obtained from a small-scale research and voluntary contributions.

1cDigitization	Workflow	Est. Avg Time
Manual Approach	Without any Interrupts	4–6 mins
	With a Chess Board to Resolve Conflicts	8–10 mins
	With the need of Player Intervention to Resolve Conflicts	12–15 mins
	Averaging a 4 round Swiss Tournament	6–8 mins
Presented Approach	Text Box Extraction & Autonomous Post-Processing	<1 min
	Text Box Extraction & Semi- Autonomous Post-Processing	1–2 mins
	Four-Corner-Marking & Autonomous Post-Processing	1–2 mins
	Four-Corner-Marking & Semi- Autonomous Post-Processing	2–3 mins

## Data Availability

The Handwritten Chess Scoresheet or HCS dataset can be accessed at https://sites.google.com/view/chess-scoresheet-dataset or http://tc11.cvc.uab.es/datasets/HCS_1 (accessed on 21 November 2021). This dataset is public and free to use for researchers.
